# MRI-based early diagnosis: a diabetic Charcot spine case report

**DOI:** 10.1186/s12883-021-02235-3

**Published:** 2021-05-19

**Authors:** Barbara Limberger Nedel, Juliana Avila Duarte, Fernando Gerchman

**Affiliations:** 1 Radiology Unit of Hospital de Clinicas de Porto Alegre, Postgraduate Program in Medical Science: Endocrinology, Universidade Federal do Rio Grande do Sul, Porto Alegre, Brazil; 2 Radiology Unit of Hospital de Clinicas of Porto Alegre and Department of Internal Medicine, Faculdade de Medicina, Universidade Federal do Rio Grande do Sul, Porto Alegre, Brazil; 3 Division of Endocrinology and Metabolism, Hospital de Clínicas de Porto Alegre, Porto Alegre, Brazil; 4 Department of Internal Medicine and Postgraduate Program in Medical Science: Endocrinology, Faculdade de Medicina, Universidade Federal do Rio Grande do Sul, Porto Alegre, Brazil

**Keywords:** Magnetic resonance imaging, Charcot spine, Spinal neuroarthropathy, Diabetes, Case report

## Abstract

**Background:**

Spinal neuroarthropathy (SNA), also known as Charcot spine, is an uncommon aggressive arthropathy, secondary to loss of proprioceptive and nociceptive feedback from the spine. A diagnosis of SNA is frequently delayed due to the scarcity of symptoms in its early stages, leading to significant neurological deterioration. Therefore, prompt suspicion of the disease is critical to providing better outcomes. This case assembles two rare characteristics of SNA: diabetic aetiology and a precocious time of diagnosis, and aims to highlight the magnetic resonance imaging (MRI) findings that allowed for the diagnosis.

**Case presentation:**

A 44-year-old woman, with long-term type 1 diabetes, presented with a two-month history of progressive lumbar pain, difficulty in maintaining an upright position, and discrete trunk forward-leaning. Diabetes-related vasculopathy and nephropathy were already known, and laboratory test results did not show any new abnormalities. A lumbar MRI revealed extensive signal intensity changes of the L2 and L3 vertebral bodies associated with marginal areas of enhancement and the involvement of regions adjacent to interapophyseal articulations and spinous processes from L2–L3 to L5–S1, in association with degenerative changes of the thoracolumbar spine. These findings were identified by the radiologist as suggestive of SNA. To rule out neoplastic and infectious disease, a bone biopsy at the L2–L3 level was executed. The pathology report revealed intervertebral disc material and fragments of fibrous tissue, with a complete absence of inflammatory cells. It was decided to perform a six-month MRI follow-up, which showed stability of the findings, confirming the hypothesis of Charcot spine. The patient was under clinical and radiological follow-up and did not require surgical fixation at the moment of diagnosis. After 2.5 years from the initial diagnosis, a new MRI revealed progression of the lesions with oedema and enlarged paravertebral soft tissues; these findings are compatible with the patient’s latest complaints of lumbar pain recurrence.

**Conclusion:**

To the best of our knowledge, this is the first case report of an MRI-based early diagnosis of diabetic SNA, a rare disease with nonspecific symptoms in its initial stages and a wide spectrum of differential diagnoses. The MRI findings, distinctly the involvement of both anterior and posterior spinal elements, were the key to allowing for the proper diagnosis. A precocious diagnosis, although challenging, is fundamental to providing early intervention and to preventing further neurological impairment.

## Background

Spinal neuroarthropathy (SNA) is an uncommon aggressive arthropathy, secondary to loss of proprioceptive and nociceptive feedback from the spine, usually in patients with traumatic spinal cord injury. The desensate spine presents areas of increased mobility and weight-bearing, leading to repetitive microtrauma, unregulated hyperaemia, and, ultimately, destruction of the intervertebral articulations [1]. The first report of SNA dates from 1868, generally attributed to Jean-Martin Charcot, a French neurologist whose name was later coined as the eponym of the disease: ‘Charcot spine’.

Severe neurological deterioration is common at the moment of diagnosis due to the scarcity of symptoms in SNA’s early stages. Therefore, a precocious diagnosis is very rare – only a few cases have been described in the literature, secondary generally to spinal trauma, as reported in 2019 by Abramoff et al. [2]. Another unusual feature of SNA is diabetes as an underlying cause, reported to be around 2.0% in a 2010 comprehensive review by Barrey et al. [3]. This case stands out for describing a diabetes-related early diagnosis of SNA, and aims to highlight the magnetic resonance imaging (MRI) findings that provide the basis for the diagnosis.

## Case presentation

A 44-year-old woman with a 35-year history of type 1 diabetes presented with a two-month history of progressive lumbar pain, night sweats and difficulty in keeping an erect posture. The patient did not have any history of falls or traumatic injury around the time of onset of symptoms. Diabetes-related vasculopathy and nephropathy had been previously diagnosed, and there was no other medical or familial history. Physical examination showed only discrete trunk forward-leaning. Initial blood tests did not show any abnormalities except for known hyperglycaemia and decreased renal function. To discard spinal infections, Doppler echocardiography to search for endocarditis, a blood culture, a purified protein derivative (PPD) test for tuberculosis and serological tests for brucellosis were performed with unremarkable results.

A lumbar MRI revealed extensive signal intensity changes of both anterior and posterior elements, findings indicated by the radiologist as suggestive of SNA. The MRI sequences demonstrated irregularities of the L2 and L3 vertebral body endplates with markedly hypointense lesions, both on the T1-weighted images (T1WI), due to the loss of normal bone marrow signal, and on the T2-weighted images (T2WI), due to sclerosis. The borders of the lesions presented hyperintensities on the STIR-weighted images (STIR-WI) and the T2WI, indicating oedema. The T2WI also showed enlargement of the paravertebral surrounding soft tissues, suggestive of inflammation with post-contrast enhancement, as depicted on the gadolinium-enhanced fat sat T1-weighted images (T1WI + C). The post-contrast sequences also demonstrated subtle marginal enhancement of the vertebral body lesions and the areas adjacent to interapophyseal articulations and spinous processes from L2–L3 to L5–S1, compatible with the involvement of both anterior and posterior elements. The gross morphology and height of the affected vertebral bodies were still preserved. There was an associated slight retrolisthesis of the L2 vertebral body, suggesting some grade of instability.

**Fig. 1:**
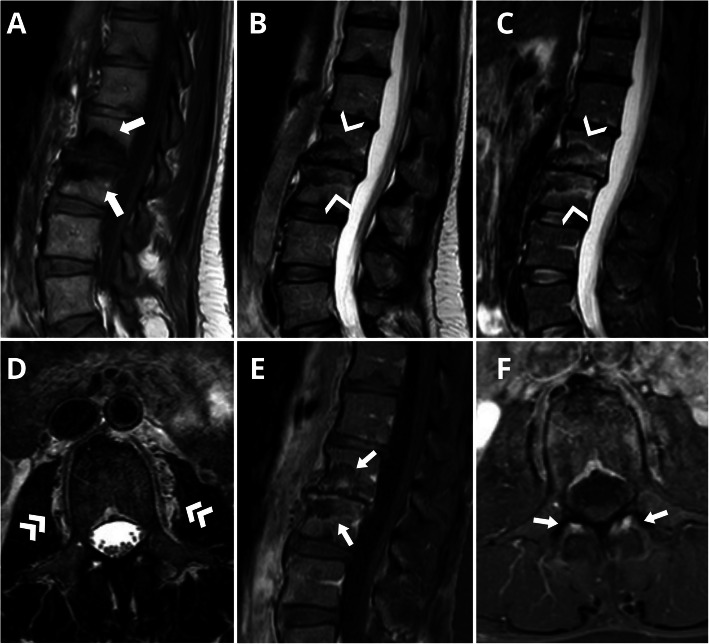
The first lumbar MRI. The sagittal T1WI (**a**) and the sagittal T2WI (**b**) show irregularities and lesions with marked hypointensity of the L2-–L3 vertebral body endplates, representing a loss of normal bone marrow signal on the T1WI (simple arrows) and sclerosis on the T2WI (open arrows). There is a slight retrolisthesis of the L2 vertebral body, suggesting some grade of instability. The gross morphology and height of the involved vertebral bodies are still preserved. There are associated degenerative changes with marginal osteophytes, important reduction of the L2–L3 intervertebral disc height and its T2WI signal, depicting disc dehydration, findings also observed at other levels of the thoracolumbar spine. The sagittal STIR-WI (**c**) demonstrate hyperintensity at the borders of the lesions, indicating oedema, a finding also observed on the T2WI in a subtler way (open arrows). The axial T2WI (**d**) show an enlargement of paravertebral surrounding soft tissues, suggestive of inflammation (double arrows). The sagittal (**e**) and axial (**f**) gadolinium-enhanced fat sat T1WI demonstrate subtle marginal enhancement of the vertebral body lesions and of the areas adjacent to interapophyseal articulations and spinous processes, from L2–L3 to L5–S1, compatible with the involvement of both anterior and posterior elements (simple arrows). The paravertebral enlarged soft tissues are also enhanced

There were associated degenerative disc changes with a collapsed L2–L3 intervertebral disc and loss of its T2WI signal, which depicted disc dehydration. These findings were also found with variable degrees at other levels of the thoracolumbar spine, more markedly at the T11–T12, T12–L1 and L1–L2 levels, in association with Schmorl nodules. There were marginal osteophytes and slight diffuse disc bulging present from the T11 to L3 levels without significant reduction of the amplitude of the corresponding neuroforamen or spinal canal. Interapophyseal degenerative changes were present at the levels of L4–L5 and L5–L1. The conus medullaris and nerve roots presented normal signal intensity and no signs of compression or pathological enhancement (Fig. 1).

**Fig. 2:**
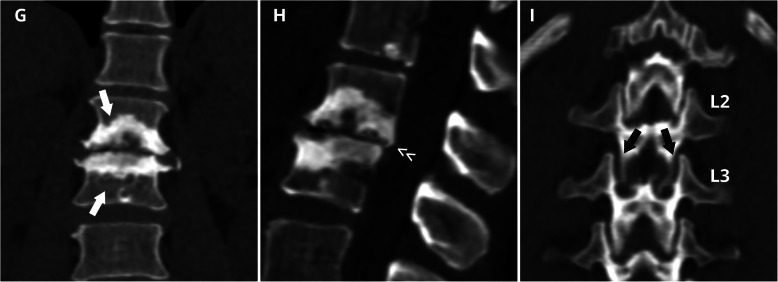
CT including lumbar spine. The coronal CT plane (G) shows severe sclerosis of the L2–L3 vertebral body endplates (white arrows) and subtle endplate irregularities due to mild destruction. Marginal osteophytes are evident. The sagittal CT plane (H) illustrates slight retrolisthesis of the L2 vertebral body, suggesting some grade of instability (double arrowheads). The L2–L3 intervertebral disc collapse is also depicted. The coronal CT plane (I) demonstrates there are no significant degenerative changes in the corresponding superior and inferior articular processes (black arrows)

In a complementary way, computed tomography (CT) images of the lumbar spine confirmed the severe grade of sclerosis of the L2–L3 vertebral body endplates, disproportional when compared to the virtual absence of destruction of these structures, which was limited to endplate irregularities. Bone-conditioning CT images also clarified the slight retrolisthesis of the L2 vertebral body. Findings related to degenerative changes, such as an L2–L3 intervertebral disc collapse and adjacent marginal osteophytes, were also observed. There were no signs of significant degenerative changes in the corresponding superior and inferior articular processes (Fig. 2).

**Fig. 3:**
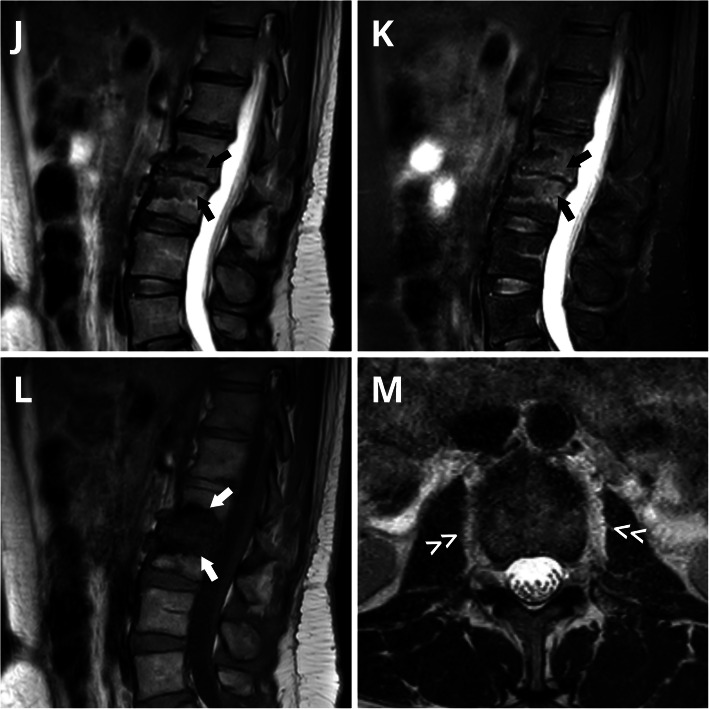
Lumbar MRI performed 2.5 years after the initial diagnosis. The sagittal T2WI (J) and the STIR-WI (K) demonstrate increased areas of hyperintensity in the L2 and L3 vertebral bodies and endplates, indicating progression of the oedema (black arrows). The sagittal T1WI (L) depict the same irregularities and lesions with marked hypointensity of L2–L3 vertebral body endplates (white arrows). The axial T2WI (M) show the progression of the enlarged paravertebral surrounding soft tissues, suggestive of inflammation (double arrows)

To exclude neoplastic and infectious disease, a bone biopsy at the L2–L3 level was executed, and the anatomopathological report revealed ‘material compatible with intervertebral disc’, without any cell infiltrate. Gram staining, direct microscopy for fungi, alcohol acid-resistant bacilli (AARB) bacilloscopy and bacteriological, mycobacterial and fungal cultures of the biopsied tissue samples were negative.

It was decided to perform a six-month MRI follow-up, which showed stability of the findings, excluding a possible infectious or malignant aetiology and corroborating the hypothesis of SNA.

After initial diagnosis in August 2018, the patient was evaluated by a neurosurgeon, who reported an agreement with the established diagnosis. Despite the severity of the majority of SNA cases, the patient’s spine was aligned and without signs of compression, therefore without indication for surgical fixation.

The patient received motor physiotherapy sessions until the beginning of the SarS-CoV-2 pandemic in Brazil, in March 2020.

The patient has been followed-up since diagnosis with clinical appointments and routine MRI exams. At the last physical examination in January 2021, 2.5 years after the diagnosis, there were no changes in the patient’s height, an important clinical parameter that changes over time in a more aggressive evolution.

Nonetheless, the last MRI in February 2021 revealed definitive progression of the findings, particularly on the STIR-WI and T2WI, represented by an increase in the vertebral body areas of hyperintensity. Additionally, there was also a slight increase in the paravertebral soft tissue component (Fig. 3).

The clinical appointment following the MRI in March 2021 occurred through remote telephone consultation, due to SarS-CoV-2 pandemic. The patient, who, until then, was under satisfactory control of the lumbar pain with simple analgesic medications, reported that the lumbar pain has worsened in the past few weeks and was no longer being controlled with medicines, symptoms compatible with the MRI findings of progression.

A new neurosurgical consultation is planned to define a complete treatment, including physiotherapy sessions, in consideration of the current limitations due to the SarS-CoV-2 pandemic.

Besides the medical conduct regarding SNA, the patient is under endocrinological follow-up for type 1 diabetes aiming for appropriate glycaemic and metabolic control.

## Discussion and conclusion

This case is notable because it assembles two rare characteristics of SNA. One unusual feature is the diabetes-related aetiology. In a comprehensive 2010 review by Barrey et al. [3] of 109 patients with SNA, only two were related to diabetes (2.0%). Indeed, few diabetic SNA cases have been previously reported, characterising a limitation of this report. Despite this, diabetes remains a very rare cause of Charcot spine, most commonly seen in neuroarthropathy of the foot and ankle.

Another atypical element of this case is the very early diagnosis. Due to the nonspecific symptoms at the initial stages of SNA, patients are frequently diagnosed with advanced disease, especially those with diabetes. The two diabetes-related SNA cases reported in the 2010 review by Barrey et al. [3] presented significant lumbar distortion, one with progressive paraparesis and one with cauda equina syndrome. In 2014, van Eeckhoudt etal [4]. described a case of diabetic SNA who presented thoracolumbar vertebral body fractures and important kyphosis. To the best of our knowledge, this is the first case report of diabetic SNA without deformity of the spinal axis or other features of advanced disease at the moment of diagnosis.

In this case, MRI findings were the key to suggesting a proper diagnosis and distinguishing SNA from its mimics. One of the main differential diagnoses of early SNA is degenerative disc disease (DDD). There were, indeed, concomitant signals of DDD, including disc space reduction, loss of the disc T2WI signal, disc bulging and marginal osteophytes, observed at other levels of the dorsal and lumbosacral spine as well. The vertebral body lesions with marked hypointensity on the T1WI and T2WI from the MRI of this patient could be mistaken for type 3 Modic changes, described in the late evolution of DDD. However, gadolinium enhancement and the T2WI/STIR-WI hyperintensity at the borders of the lesions, as well as paravertebral soft tissue enlargement with enhancement, could not be explained solely by DDD. Additionally, neuroarthropathy should be considered in patients who are at risk of neuropathy, such as our patient, who had long-term type 1 diabetes [5].

Another important differential diagnosis to consider is infectious spondylodiscitis. The exclusion of this entity was based on the negative results of laboratory tests, the pathology report and imaging evaluations. There was an absence of T2WI/STIR-WI hyperintensity within the L2–L3 intervertebral disc, an MRI feature usually observed in cases of bacterial spondylodiscitis. Moreover, the absence of progression of the findings in the six-month control MRI corroborated the hypothesis of a noninfectious aetiology, considering no antimicrobial treatment was instituted. Although SNA cases typically deteriorate with destructive lesions over time, we believe our patient’s findings of six-month stability are related to an extremely early presentation of SNA.

A bone biopsy was performed primarily to discard malignant and infectious disease, another important differential diagnosis of SNA. The anatomopathological report stated there was ‘material compatible with intervertebral disc without cell infiltrates’, a surprising result considering the surrounding extensive inflammatory abnormalities shown in the MRI. We requested a review of the pathology slides, and the pathologist has confirmed that the sample represented intervertebral disc material and fragments of fibrous tissue, with a complete absence of inflammatory cells. We believe that the absence of inflammatory cells in the bone biopsy results can be explained by the significant collapse and dehydration of the L2–L3 intervertebral disc. As stated by Moore et al. [6], discs of the human spine are the largest non-vascularised structures in the body, and disc nutrition and metabolic exchange are almost totally reliant on diffusion of essential solutes across the endplates. Therefore, a significant reduction in the water content of the disc would impede the exchange of nutrients or the arrival of cell infiltrates.

Additionally, this case stands out as an illustration of early Charcot spine findings in the two imaging methods, MRI and CT. Considering that MRI is not available worldwide and that CT is frequently the preferred initial method for lumbar pain evaluation, recognition of the CT imaging pattern of SNA may be crucial to suspecting and proceeding with further investigation. Noteworthy is the severity of the changes involving the vertebral plateaus and the vertebral bodies of L2 and L3, while the signs of DDD at the other levels were much less important and mild. From an educational perspective, we believe that the correlation between both methods may provide a better understanding of the findings.

Finally, the most recent patient’s MRI examination, performed 2.5 years after initial diagnosis, revealed definitive progression of the previously described findings, particularly on STIR-WI and T2WI, represented by an increase in the hyperintensity of the vertebral bodies and endplates. Additionally, there was also a slight increase in the paravertebral soft tissue component. These findings corroborate the hypothesis of an early case of SNA with subtle progression over time that became more evident from long-term follow-up. Unfortunately, this MRI was performed without intravenous gadolinium-based contrast agent injection since the patient was not complaining of pain when the exam was ordered.

One of the main limitations of this report is the absence of additional imaging of other joints, such as hips, knees and feet. An extended radiological evaluation could add to the discussion and patient care, considering that neuroarthropathy in type 1 diabetes patients may not be limited to the spine, even in asymptomatic patients. In actuality, complementary images were requested after last virtual consultation, but the patient was unable to undergo the examinations because a family member of hers was hospitalised with COVID-19.

We sought to highlight the radiologist’s central role in the diagnostic workup and to propagate the imaging clues suggestive of SNA. Early diagnosis of Charcot spine, though challenging, is fundamental to providing precocious intervention, whether through surgery or aggressive conservative treatment and to prevent further neurological deterioration.

SNA has a wide spectrum of differential diagnoses, including degenerative disk disease, spondylodiscitis, haemodialysis-related spondyloarthropathy, pseudarthrosis, SAPHO syndrome and Andersson lesions. Careful attention to some specific imaging clues, especially the involvement of both anterior and posterior spinal elements, may help the physician set the proper diagnosis. Other MRI features suggestive of SNA include vacuum phenomenon within the disk (due to excessive motion), malalignment, paraspinal soft-tissue masses or fluid collections containing bone debris.

## Data Availability

Data sharing not applicable to this article as no datasets were generated or analysed during the current study.

## Abbreviations

SNA: Spinal neuroarthropathy
MRI: Magnetic resonance imaging
